# Combination of Rapamycin, CI-1040, and 17-AAG Inhibits Metastatic Capacity of Prostate Cancer via Slug Inhibition

**DOI:** 10.1371/journal.pone.0077400

**Published:** 2013-10-10

**Authors:** Guanxiong Ding, Chenchen Feng, Haowen Jiang, Qiang Ding, Limin Zhang, Rong Na, Hua Xu, Jun Liu

**Affiliations:** Department of Urology, Huashan Hospital, Fudan University, Shanghai, China; University of Kentucky College of Medicine, United States of America

## Abstract

Though prostate cancer (PCa) has slow progression, the hormone refractory (HRCP) and metastatic entities are substantially lethal and lack effective treatments. Transcription factor Slug is critical in regulating metastases of various tumors including PCa. Here we studied targeted therapy against Slug using combination of 3 drugs targeting 3 pathways respectively converging via Slug and further regulating PCa metastasis. Using *in vitro* assays we confirmed that Slug up-regulation incurred inhibition of E-cadherin that was anti-metastatic, and inhibited Bim-regulated cell apoptosis in PCa. Upstream PTEN/Akt, mTOR, Erk, and AR/Hsp90 pathways were responsible for Slug up-regulation and each of these could be targeted by rapamycin, CI-1040, and 17-AAG respectively. In 4 PCa cell lines with different traits in terms of PTEN loss and androgen sensitivity we tested the efficacy of mono- and combined therapy with the drugs. We found that metastatic capacity of the cells was maximally inhibited only when all 3 drugs were combined, due to the crosstalk between the pathways. 17-AAG decreases Slug expression via blockade of HSP90-dependent AR stability. Combination of rapamycin and CI-1040 diminishes invasiveness more potently in PCa cells that are androgen insensitive and with PTEN loss. Slug inhibited Bim-mediated apoptosis that could be rescued by mTOR/Erk/HSP90 inhibitors. Using mouse models for circulating PCa DNA quantification, we found that combination of mTOR/Erk/HSP90 inhibitors reduced circulating PCa cells *in vivo* significantly more potently than combination of 2 or monotherapy. Conclusively, combination of mTOR/Erk/Hsp90 inhibits metastatic capacity of prostate cancer via Slug inhibition.

## Introduction

Prostate cancer (PCa) is a common neoplasm, which still ranks high as the leading cause of death among urological malignancies, and stays the second leading cause of cancer deaths in males [1]. Although early detection of PCa has improved clinical outcome, metastatic PCa and hormone refractory prostate cancer (HRPC) remain one of the most challenging clinical problems，which leads to a late-stage event with a poor prognosis. PCa has a striking tendency to metastasize to bone. The 5-year survival rate of primary prostate cancer approaches 100%, and however declines to 33% if bone metastasis is diagnosed [2]. Androgen-deprivation therapy (ADT) is currently suggested for men who are diagnosed with or develop advanced or metastatic PCa after local treatment [3]. Unfortunately, resistance to ADT eventually emerges, usually manifesting as tumor regrowth associated with an increase in the serum prostate-specific antigen (PSA) levels, and in the case of HRPC, fatal outcomes is usually associated [4,5]. Traditional therapeutic strategies (chemotherapy and radiotherapy) are often associated with unsatisfying outcomes in this population. Therefore, targeted therapy has emerged as a promising alternative modality for patients with metastatic PCa or HRPC. Development of more effective therapeutic interventions based on the molecular studies by which tumors develop resistance to therapeutic drugs is thus an urgent need. Recent work has been aiming at identifying key molecules involved in metastasis as therapeutic targets. 

Slug (Snai2) is a member of the Snail family, which is a zinc-finger transcription factor. It is also one of the vertebrate-specific genes associated with Snail. It has been confimred in a number of in vitro studies that Slug is critical to metastasis and invasion ability of cancer cells [6,7]. Studies have also shown that Slug expression may be increased in certain organs (breast and stomach tumor tissue), but decresed in others (such as colon, ovary and esophagus normal tissues). Our previous study shows that Slug protein is highly expressed in the prostate cancer tissues, and that Slug protein is expressed in PC-3, LNCaP, DU-145, and 22RV1 PCa cell lines. Its expression may be subjected to regulation at transcription or post-translation modification. We have also found that Slug protein is highly expressed in tumor samples but not in normal prostate tissue [8]. Therefore, in the current study we aim at studying the how Slug is implicated in the metastatic capacity of PCa and at testing the efficacy of targeted therapy against Slug related pathways.

## Materials and Methods

### Reagents

Rapamycin, CI-1040, 17-AAG, DHT (0.1 mg/mL) and primary antibodies of Slug (rabbit), pS6 (pSer235/236, rabbit), pAkt (pSer473, rabbit), PTEN (rabbit), HIF-1α (mouse), HSP90 (rabbit), AR (rabbit), and β-actin (mouse) were purchased from Sigma-Aldrich, Munich, Germany. Antibodies of pErk (pThr202 / pTyr204, rabbit), and Erk (rabbit) were purchased from Cell Signaling Technology (Danvers, MA). Secondary antibodies were purchased from Santa Cruz, USA. The SuperSignal West Pico chemiluminescent substrate kit (Thermo Scientific, IL) was used. Human Slug and control siRNAs were purchased from Santa Cruz.

### Cell culture

Human DU145, PC-3, LNCap and 22RV1 prostate adenocarcinoma cell lines were commercial and were purchased from Cell Bank of Chinese Academy of Sciences (Shanghai, China). LNCap and 22RV1 cells were cultured in RPMI 1640 media (PAA, Germany) with 10% fetal bovine serum (FBS) (PAA). DU145 and PC-3 cells were cultured in Ham’s F-12 media (Gibco, NY) with L-glutamine (300mg/L, NaHCO_3_ 1.5g/L) and 10% FBS. Cells were incubated with 5% CO_2_ at 37°C.

### Western blotting

Total protein of lysates was extracted and purified. Equal protein amount of 25μg was loaded onto 10% sodium dodecyl sulphate polyacrylamide gel for electrophoresis. Gels were subsequently transferred to nitrocellulose membrane. The membranes were blockaded for 1 h with 5% non-fat milk. Primary antibodies of Slug, pS6, pAkt, PTEN, pErk, Erk, HIF-1α, HSP90, AR, and β-actin were then added and membranes were incubated at 4°C overnight. Corresponding secondary antibodies were applied followed by horseradish peroxidase application.

### Migration and invasion assay

In vitro migration assays were performed as previously described. Briefly, cells were seeded in the top chamber of the 8.0μm pore size cell culture inserts that were either coated or uncoated with matrigel for migration and invasion assays, respectively. Then the inserts were placed in a 24-well plate filled with medium with 5% fetal bovine serum. Cells that penetrated to the underside surfaces of the inserts were fixed and stained with the Diff-Quick (Fisher Scientific, Pittsburgh, PA) method and were counted under the microscope. The mean of three high power fields for each condition run in triplicates was calculated.

### Realtime PCR

Total RNA was extracted with RNAiso reagent (TaKaRa, Dalian, China). After concentration was determined with Thermo Nanodrop 1000 spectrophotometer, RNAs were converted to cDNAs with PrimeScript RT Reagent Kit (TaKaRa) under the condition of 37°C, 15 min; 85°C, 5 sec. Forward and reverse primers of Slug, E-cadherin, and internal control GAPDH (glyceraldehyde-3-phosphate dehydrogenase) were synthesized (Invitrogen) (Tab. 1) as follows sequences: for human E-cadherin, sense, 5’-ACA GCC CCG CCT TAT GAT T-3’ and antisense, 5’-TCG GAA CCG CTT CCT TCA-3’; for Bim, sense, 5’-GGT CCT CCA GTG GGT ATT TCT CTT-3’ and antisense, 5’-ACT GAG ATA GTG GTT GAA GGC CTG G-3’; for Caspase-3, sense, 5’-GAC AGA CAG TGG TGT TGA TGA TGA C-3’ and antisense, 5’-GCA TGG CAC AAA GCG ACT GGA T-3’; for Slug, sense, 5’-TTC GGA CCCACA CAT TAC CT-3’ and antisense, 5’-GCA GTG AGG GCA AGA AAA AG-3’; for GAPDH, sense, 5’-ATG GAA ATC CCA TCA CCA TCT T-3’ and antisense, 5’-CGC CCC ACT TGA TTT TGG-3’. Samples were processed with SYBR Green Premix Ex Taq (TaKaRa) in 20μl system on ABI 7500n (Applied Biosystem, Forster City, CA). Samples were run at 95°C, 30 sec and were amplified for 40 cycles (95°C, 5 sec; 60°C, 34 sec). For each sample, the average value of threshold cycle was normalized to GAPDH level with the formula, 2^-ΔΔCt^. Results were thus presented by expressional folds over control.

### Xenograft and intravenous tumor model

Male BALB/c athymic nude mice at 6 weeks of age (Vital River, Beijing, China) were bred in licensed SPF (special pathogen-free) grade laboratory. All mice underwent orchiectomy for androgen deprivation. A total of 2 ×10^6^ cells (DU145, PC-3, LNCap, 22RV1 respectively) in 100μl of PBS were injected subcutaneously at both flanks of each mouse. Vehicle control group was given 100μl PBS. Tumor growth was monitored and peripheral blood was collected at 1 mo. For intravenous injections, 2 × 10^5^ cells were injected into the lateral tail vein and peripheral blood was collected at 24 h. All protocols were approved by Fudan University animal ethics committee. 

### Cell death detection

Cell death was detected using MTT assay, a method that was well established. Briefly, the cells were firstly given different treatments, and then exposed to 400 μM H_2_O_2_ for 4 h. In control groups, the RIN-mβ cells were only treated with 400 μM H_2_O_2_ and corresponding drug, respectively. At the end of the treatments, the media were removed and the cells were stained and measured at 495 nm using an Autoplate reader (Bio-Tek, USA). The treatment with 400 μM H_2_O_2_ for 4 h could induce apoptosis of approximately half of PCa cells (~50%) and the dose was thus selected as optimal condition.

### Circulating tumor DNA detection

Mouse blood (0.5 mL) was collected at indicated times by intraocular bleed, and red blood cells were lysed before DNA extraction. The Quick-gDNA MiniPrep kit (Epigenetics, USA). Briefly, 400 μl of lysis buffer were added to 100 μl of plasma. After vigorous vortex, all contents were moved to columns and were centrifuged. Pre-wash buffer was then added followed by another centrifuge and addition of wash buffer. DNAs were finally eluted with DNA elution buffer and were quantified by using TaqMan-chemistry based realtime PCR assays as aforementioned for human Slug and E-cadherin expressions.

### Statistical analysis

One-sample and Two-sample t-tests, and Mann-Whitney test were used for in vitro and in vivo studies. A p value or <0.05 was accepted as statistical significance. Data were presented as mean ± standard deviation (SD).

## Results

### Androgen promotes metastatic potential of PCa via up-regulation of Slug

**Figure 1:**
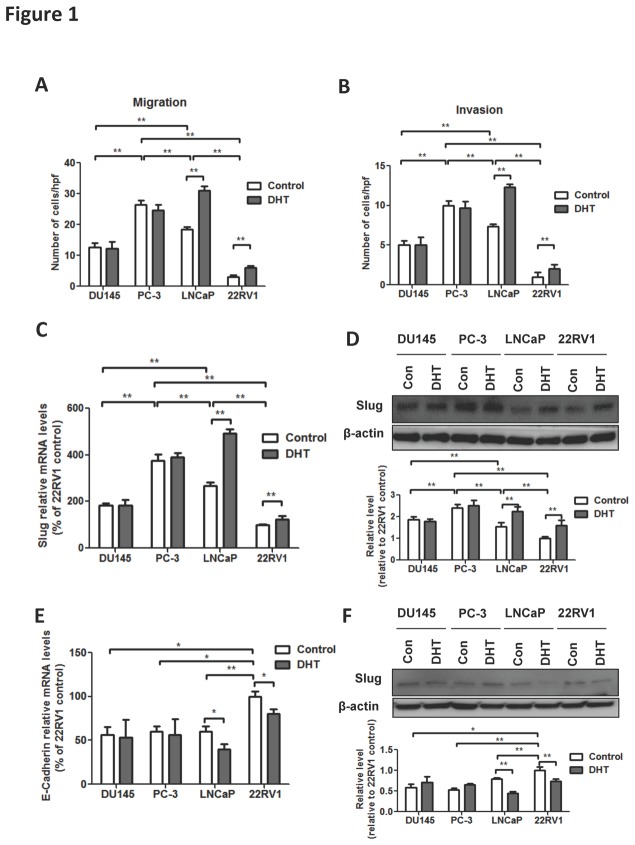
In vitro assays profiling the aggressiveness of the cell lines in response to androgen treatment are carried out. Migration (A) and invasion (B) assays performed in 4 PCa cell lines treated with dihydrotestosterone (DHT) and control. Levels of mRNA (C) and protein (D) of Slug in PCa cell lines treated with DHT or control. Levels of mRNA (E) and protein (F) of E-Cadherin in PCa cell lines treated with DHT or PBS (control). (Error bar = standard deviation; n = 3 for each group; *p < 0.05; **p < 0.01).

Prostate cancer fed on androgen in the majority of cases and transition to the hormone refractory phenotype (HRPC) usually required long-term androgen deprivation therapy. We thus investigated here whether Slug was implicated in the androgen-mediated metastatic pathway in prostate cancer. By applying dihydrotestosterone (DHT) at 1 nM to 4 PCa cell lines with different traits, we found that cells were different for their inherent migrating capacity with different sensitivity to androgen (Figure 1A). The PC-3 cells were most inherently invasive, and LNCaP and 22RV1 were more sensitive to androgen stimulation with significant increases in metastatic profile. Similar pattern of change in invasiveness was also observed in the invasion assay, confirming the profiles aforementioned (Figure 1B). To elucidate whether Slug was implicated in the hormone-related metastasis/invasion enhancement, we explored the mRNA and protein levels in those cells. Accordingly, expression of Slug was significantly increased significantly in LNCaP and 22RV1 cells that were hormone sensitive in response to DHT (Figure 1C). The expression change in mRNA also corresponded to changes in protein level, as revealed in immunoblotting (Figure 1D). E-Cadherin, a protein crucial for cell-cell adhesion has been established to effect down-stream of Slug, inhibiting epithelial-mesenchymal transition (EMT) and subsequent cell evasion. We further studied whether activation of Slug by androgen incurred inhibition of E-Cadherin to promote cell migration and invasion. In line with previous assays, both mRNA and protein levels of E-Cadherin were decreased following DHT addition in cells sensitive to androgen (Figure 1E, F). Conclusively, here we demonstrated the differences of androgen sensitivities of 4 PCa cell lines and revealed that cells sensitive to androgen stimulated had increased ability for migration and invasion via elevated Slug and decreased E-Cadherin activity. Thus, for cells insensitive to androgen, which was supposed to be hormone-deprivation resistant, there should be pathways on which the migration relied and to which the invasion could be targeted.

### Combination of rapamycin and CI-1040 diminishes invasiveness of PCa

**Figure 2:**
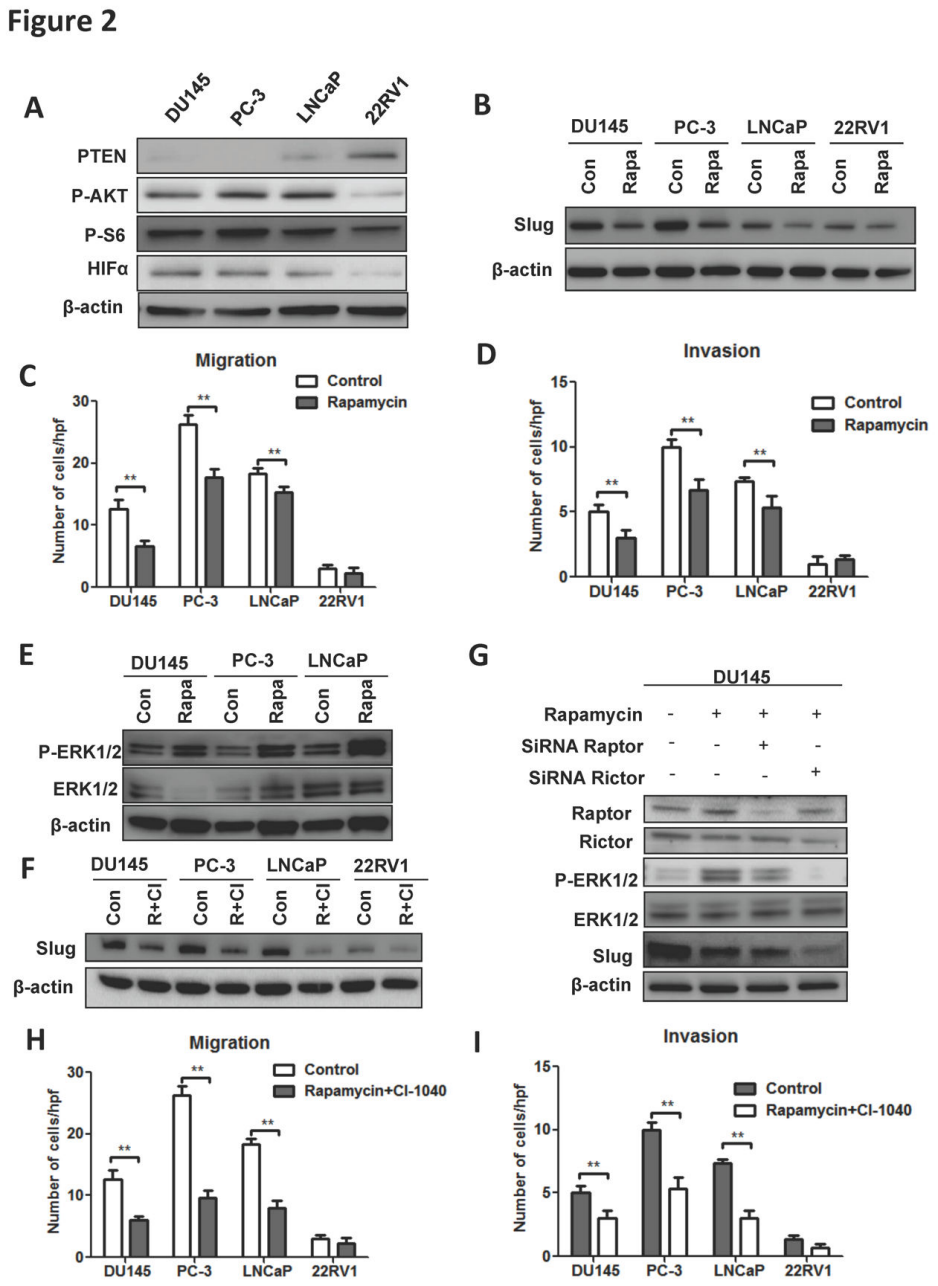
Cells were tested for the changes in mTOR and HIF pathways. Basal levels of PTEN, phosphorylated Akt and S6, and HIF1α in the PCa cell lines indicated by Western blot (A). Change of Slug level in PCa cells treated with rapamycin over control (B). Migration (C) and invasion (D) assays in PCa cell lines with or without rapamycin treatment. Activation of Erk 1/2 pathway in PCa cells responding to rapamycin treatment (E). Combination treatments of rapamycin (R) and CI-1040 (CI) in PCa cell lines and the effect on Slug (F). Combinations of rapamycin and Raptor/Rictor siRNAs and the effects on ERK and Slug in DU145 cells (G). Migration (H) and invasion (I) assays in PCa cell lines with or without combination treatment with rapamycin and CI-1040. (Error bar = standard deviation; n = 3 for each group; *p < 0.05; **p < 0.01).

Hyperactivity of PTEN/Akt/mTOR pathway has been proven to be pro-tumorigenic in a variety of cancers including the tumourigenesis of PCa, in which Slug was known to be a down-stream effector of hypoxia inducible factor-1α (HIF-1α). We then studied whether inhibition of mTOR decreased the metastatic potential of PCa via Slug. We detected major loss of PTEN in DU145 and PC-3 cells, minor loss in LNCaP cell compared to 22RV1 cell. The down-stream surrogates for Akt, mTOR, and HIF-1α activities were all increased in the PTEN-null cells, indicating a switch to mTOR pathway for androgen-insensitive phenotypes (Figure 2A). Addition of rapamycin at 10nM decreased Slug levels in all cell lines but the basal level of slug appeared higher in DU145 and PC-3 cells (Figure 2B). Rapamycin at the same dose inhibited the migration and invasion of cells with PTEN loss by ~30% and did not affect 22RV1 (Figure 2C, D). As mTOR inhibition could entail feedbacks that were on the contrary pro-tumorigenic, we examined the activity of ERK1/2, which was reported to play alternative role in regulation of slug expression in PCa previously. Strikingly, ERK1/2 activity was elevated in all 3 cell lines with PTEN loss following rapamycin application, indicating a bypassing existing circumventing mTOR towards Slug (Figure 2E). We thus tested whether the combination of rapamycin and CI-1046, an ERK1/2 inhibitor, which is in the clinical trial could further decrease the metastatic profile in these cells. Western blotting showed that the combination of drugs substantially reduced the Slug level in all PCa cell lines (Figure 2F). We then tested whether rapamycin effected via blockade of mTORC1 and exerted its downstream effect via ERK and on Slug. Treating DU145 cells with different combinations of rapamycin, Raptor and Rictor siRNAs, we found that, though a complete blockade of both mTOC-1 and -2 elements resulted in maximum inhibition of ERK and Slug, the inhibitory effect of rapamycin was possibly via mTORC1-ERK-Slug pathway (Figure 2G). The synergistic effect of combination of 1nM of rapamycin and 0.5μM of CI-1040 was shown in Figures 2H, I. There were greater inhibition of migration and invasion capacity in cells with PTEN loss reaching ~50% compared to monotherapy of rapamycin. In conclusion, androgen-insensitive PCa were generally mTOR hyperactive due to PTEN loss. Monotherapy with rapamycin however entailed ERK activation, which activated Slug as a bypass. Combination of mTOR and ERK inhibitors conferred better effect than monotherapy.

### 17-AAG decreases Slug expression via blockade of HSP90-dependent AR stability

**Figure 3:**
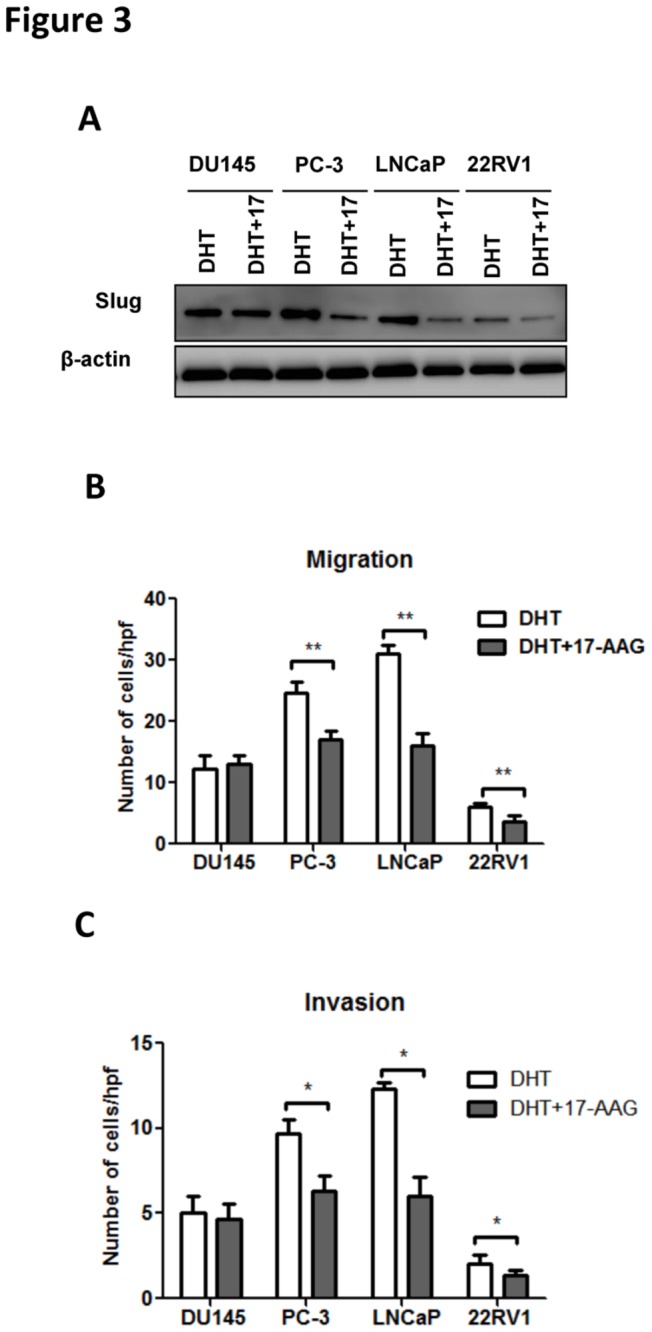
PCa cells are tested for combination of DHT and 17-AAG. Slug levels in PCa cells treated with DHT or combination of DHT and 17-AAG (A). Migration (B) and invasion (C) assays in PCa cell lines with or without combination treated with DHT or combination of DHT and 17-AAG. (Error bar = standard deviation; n = 3 for each group; *p < 0.05; **p < 0.01).

Albeit hormone deprivation had long-lasting effect on PCa, it could ensue a series of systemic adverse events. As androgen exerted its effects via binding to its receptor (AR), which induced Slug activation, we tested whether the targeted therapy against Slug pathway could be aiming at AR. It has been established that heat shock protein 90 (Hsp90) is required for the stability of proteins including AR. We then studied whether use of Hsp90 inhibitor 17-AAG could diminish the effect of androgen on metastatic profiles of PCa via inhibition of Slug. Cells were treated either with DHT alone or with combination of 300nM of 17-AAG. As expected, combination of 17-AAG blockaded Slug activity more apparently in hormone sensitive cell lines (Figure 3A). Additionally, inhibition of migration and invasion was not only observed in androgen sensitive cell lines but in PC-3 cells as well (Figure 3B, C). To sum up, blockade of Hsp90 destabilized AR leading to decreased effect of androgen in promoting cell migration and invasion. Crosstalk effect of 17-AAG could exist as it also exerted effect in androgen insensitive cells.

### Slug inhibits Bim-mediated apoptosis that can be rescued by mTOR/Erk/HSP90 inhibitors

**Figure 4:**
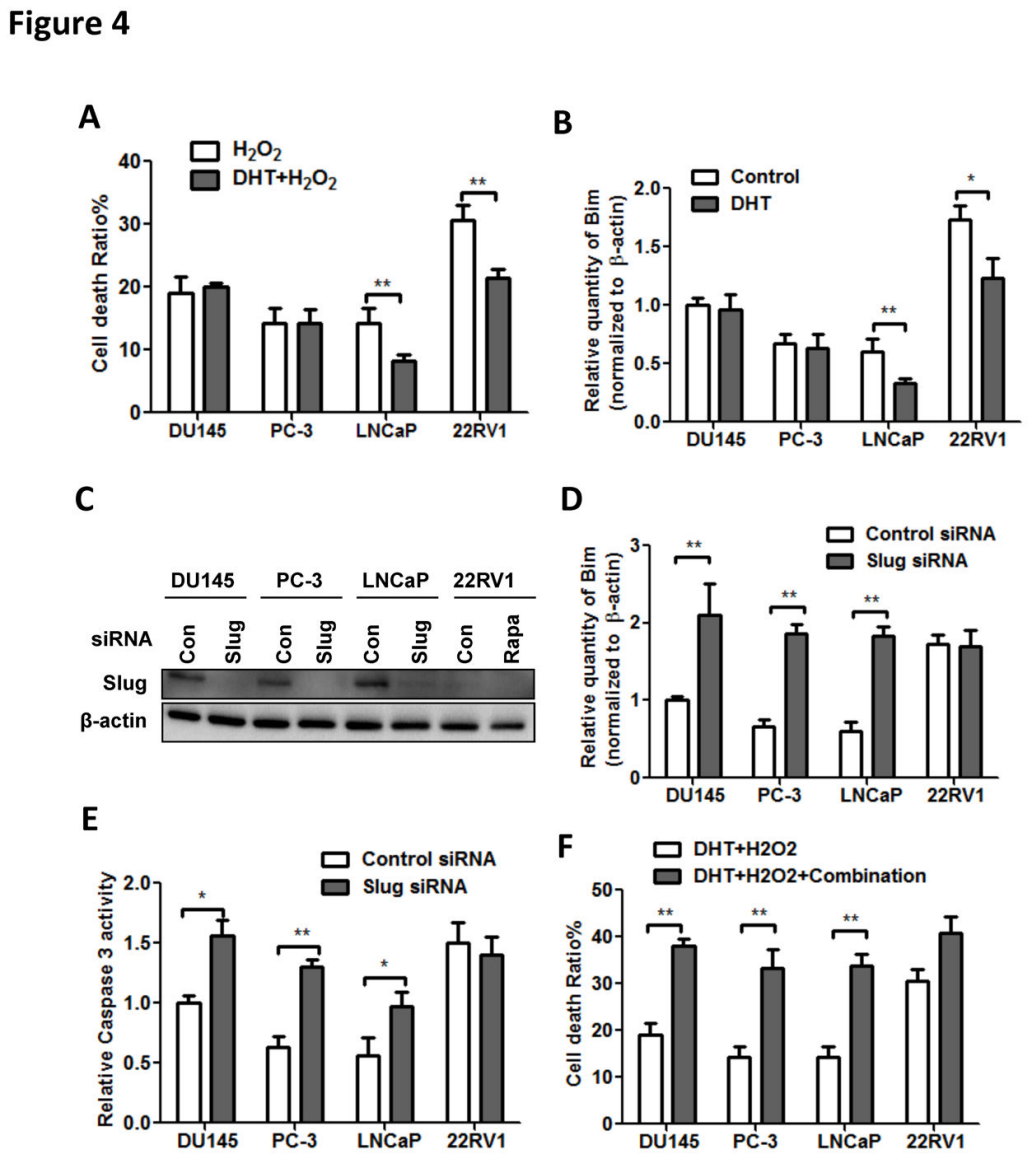
Cells were tested for resistance to ROS with combined treatment. Cell death in PCa cells treated with DHT in response to ROS simulated by H_2_O_2_ (A). Expression level of Bim in PCa cells treated with DHT (B). Knockdown of Slug with siRNA (C) and the mRNA level of Bim in PCa cell lines following Slug knockdown (D). Change of Caspase 3 mRNA level in PCa cells following Slug knockdown (E). Effect of Combination of rapamycin, 17-AAG, and CI1040 in cell death of PCa cells treated with DHT and H_2_O_2_. (Error bar = standard deviation; n = 3 for each group; *p < 0.05; **p < 0.01).

Thus far, we have established effects of targeting 3 Slug-related pathways with profound effects in inhibiting metastatic profiles. As Slug was also responsible for its down-stream anti-apoptotic effects in tumor cells, we examined whether the combination of Slug-targeting drugs conferred pro-apoptotic effects. We first mimicked Reactive oxygen species (ROS) using H_2_O_2_, and found that cells with androgen insensitivity were more resistant to ROS and androgen could rescue apoptosis in hormone sensitive cell lines (Figure 4A). As Bim was responsible for the Slug-mediated anti-apoptosis, we further determined the expression of Bim in all cells treated with DHT. Expression of Bim remained unchanged in androgen insensitive cell and decreased significantly in androgen sensitive cells (Figure 4B). To better characterize the role of Slug in anti-apoptosis, we applied siRNA against Slug expression in all cell lines (Figure 4C). The inhibitory effect was prominent except for 22RV1 cell, in which the Slug activity was constitutively low, accounting for its lowest inherent metastatic capacity (Figure 1A). We further tested the Caspase-3 activity following Slug knockdown. Expression of Caspase-3 was significantly decreased in 3 cell lines with hyperactive Slug and remained unchanged in 22RV1 cells (Figure 4E). We then applied the combination of rapamycin, CI-1040, and 17-AAG to the untransfected cells to test the synergistic effects. Strikingly, the combination rescued the anti-apoptotic effect of Slug initiated by androgen and ROS in all cell lines, in which statistical significances were obtained for DU145, PC-3 and LnCaP cells (Figure 4F). In all, Slug conferred anti-apoptotic effects in PCa cells via inhibition of Bim, pathway more likely to be activated in the hormone sensitive cells with the presence of androgen. A pan-blockade of the mTOR/ERK/Hsp90 appeared to be in inhibiting Slug as well as it s down-stream activity.

### Combination of mTOR/Erk/HSP90 inhibitors reduces circulating PCa cells *in vivo*

**Figure 5:**
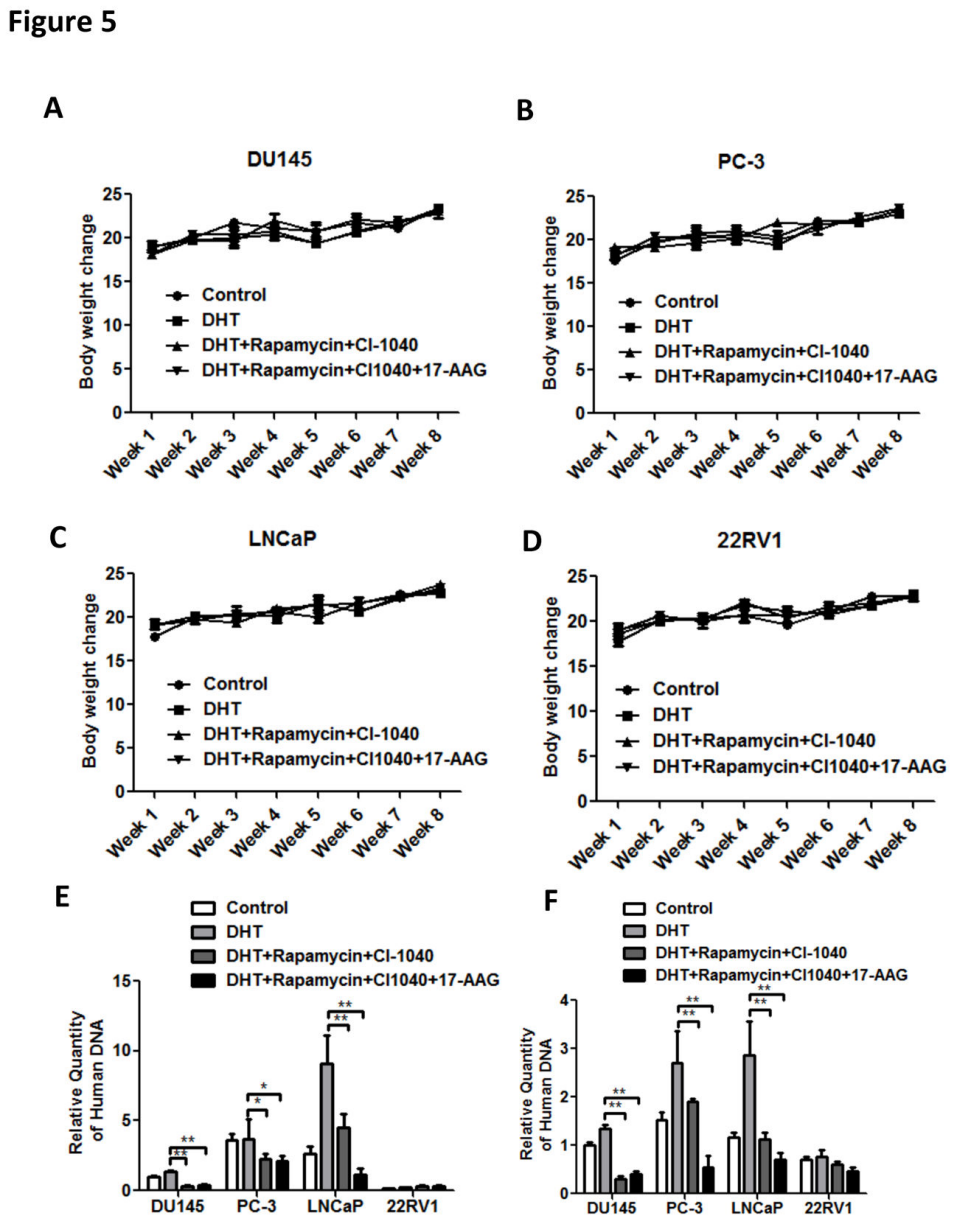
Drug toxicities in terms of mouse body weights are tested. Trend of weight change over the treatment period in all 4 PCa cell xenograft mice (A-D). Circulating PCa DNA quantification in xenograft mouse models implanted with 4 PCa cell lines and treated with different drug combinations (E). Circulating PCa DNA quantification 24 h after venous injection of PCa cells in mice that receive single treatment of drug combinations (F). (Error bar = standard deviation; n = 6 for each group; *p < 0.05; **p < 0.01).

PCa are much more prone to bone metastasis than other organs, for which hematogenous metastasis is prerequisite. We thus established animal models to test the effect drug combination in vivo. In lieu of establishing the bone metastatic model, which was hard to track the minimally metastatic lesions, we sought to detect circulating tumor cells, profiling the metastatic capacity of the primary tumor. We first established xenograft models with the 4 cell lines respectively. In order to maximally normalize androgen level, we performed orchiectomy in all mice and injected 200μg of DHT in sesame oil-ethanol subcutaneously every other day. Mice were injected with 200μl of a 1.2mg/ml solution of rapamycin daily (5 days per week). Mice were also treated twice a day by intraperitoneal injection (IP) of 100mg/kg CI-1040 whilst 17-AAG (80mg/kg/d in sterile corn oil) was delivered by 1 IP injection per day. During the treatment, the body weights of all mice were monitored for toxicity monitoring. Figures 5A, B, C, D showed the weights of mice implaneed with different PCa cell lines under different treatments. There were no significant changes between treatments and control groups, indicating non-toxic effects of the treatments. After the blood had been harvested the quantitative realtime PCR revealed that LNCaP cells were the most potent in metastatic capacity whilst 22RV1 cells hardly metastasized. The triple therapy effectively curbed tumor evasion into the circulating system in the rest 3 cell lines compared to either 2 drugs or hormone deprivation alone (control) (Figure 5E). We next aimed to study whether the combination affected anchoring capacity of the circulating tumor cells and we thus injected tumor cells into the mouse circulation. After treatment with a single dose of rapamycin and 17-AAG, and twice injection of CI-1040 as aforementioned, blood was harvested at set time point. As shown in Figure 5B, the triple therapy significantly reduced circulating tumor cells. Combination of rapamycin and CI-1040 achieved similar effects compared to androgen deprivation. Taken together, we not only demonstrated that the triple medication of mTOR/ERK/Hsp90 inhibition reduced metastatic capacity of the tumor, but also showed that such targeted therapy overwhelmed the sensitivity to androgen of the cells. The inhibitory was consistent in cells with or without hormone sensitivity and regardless of the presence of androgen (Figure 5F).

## Discussion

**Figure 6:**
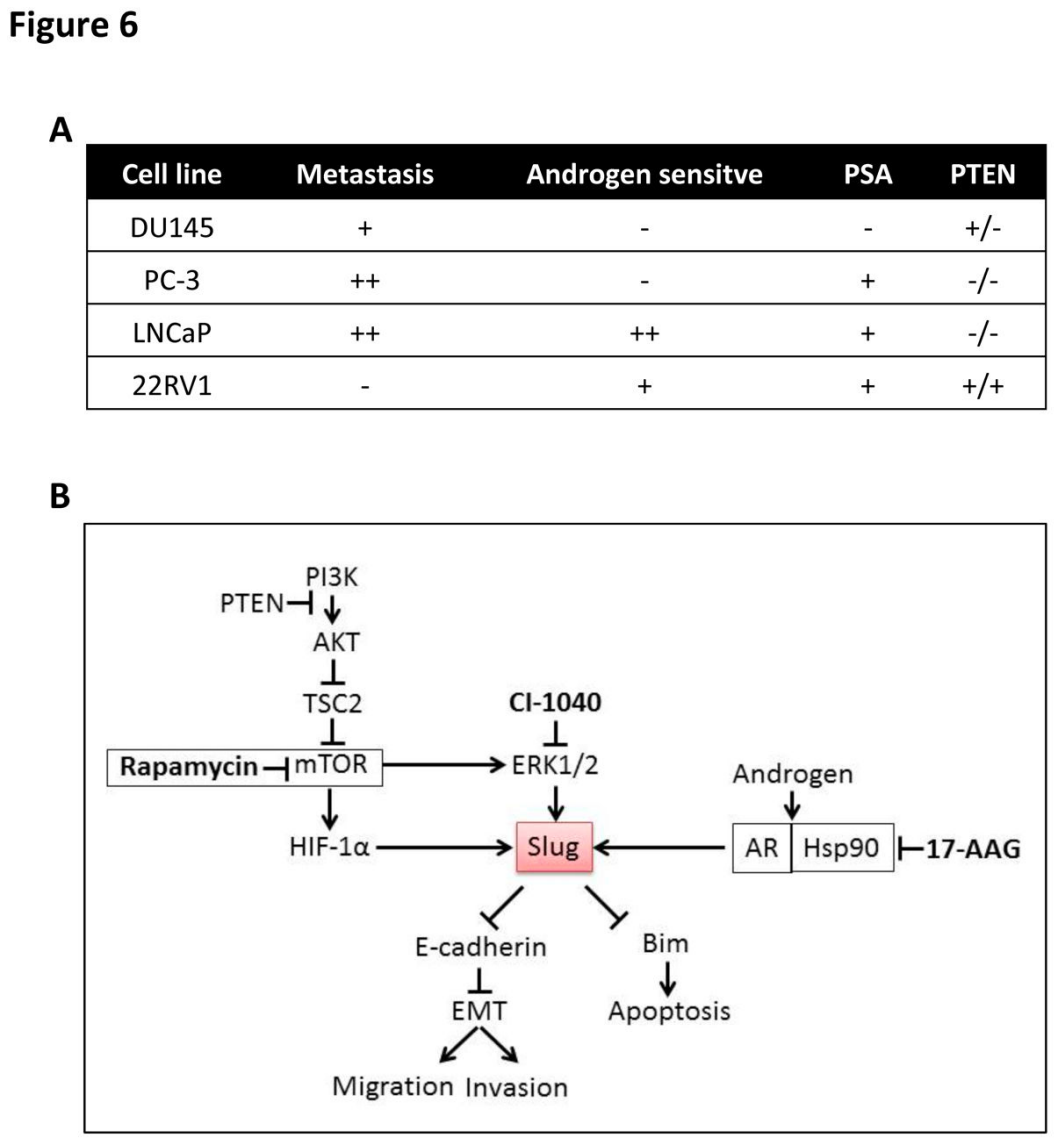
Traits of the 4 PCa cell lines used are summarized. Profiling of the PCa cells is based on this study based on our results and literature reports (A). The schematic pattern showing how the combination of drugs effect via targeting the Slug-related pathway where Slug is situated in the hub (B).

Combating PCa remains a major health problem, especially for patients with advanced-stage disease, for whom targeted therapy may bring hope. Here we have demonstrated that combined targeted therapy against Slug is effective in metastatic PCa models. Based on previous literature and our results, we may conclude the different traits of the PCa cells, which may benefit future studies for appropriate model establishments (Figure 6A). More importantly, we may propose that Slug is situated at the convergence of the mTOR/Erk/HSP90-AR pathway and is therefore responding more to the combination rather than monotherapy (Figure 6B). By blockading the up-stream of Slug, the inhibition of metastatic capacity should directly benefit from E-cadherin activation. E-cadherin is a calcium-dependent cell-cell adhesion protein and functions as a tumor suppressor. The loss of E-cadherin expression or function is a common event in tumor progression [9]. Down-regulation of E-cadherin is the key target of epithelial to mesenchymal transition (EMT) modulators, which is known to dismantle cadherin-medicated cell**–**cell junctions and essential for embryonic development, cancer progression, and chemotherapy resistance [10,11]. Because of its crucial role in EMT, E-cadherin requires a tight control in cancer. So in most scenarios of cancer, E-cadherin expression is suppressed at the transcriptional level. Various pro-tumorigenic pathways, such as MAPK/Erk, PI3K/Akt/mTOR, and Hsp90/AR, participate in EMT regulation [12,13], and all share a common end point: the activation of a series of transcription factors that directly repress E-cadherin. Several transcription factors have been identified that suppress E-cadherin including Snail, Slug, Twist and ZEB1 via their interaction with the E-box binding site in the E-cadherin promoter [14,15].

The PI3K/Akt/mTOR signaling pathway is well documented to be frequently deregulated and serves as an oncogenic pathway in several kinds of carcinomas [16]. It has been well established that the mTOR signaling pathway regulates protein synthesis in response to various growth factors and consequently affects both cell survival and cell proliferation [17]. In the present study, the inhibition of PI3K/Akt/mTOR signaling, by rapamycin, partially abolished HIF-1α-induced Snail and Slug expression levels at short duration, suggesting Slug expression can be regulated by PI3K/Akt/mTOR signaling [18,19]. The activation of PI3K/Akt signaling has been found to stimulate Snail and Slug expression via GSK-3β/β-catenin signaling and to subsequently down-regulate E-cadherin in different cellular contexts [20]. The current data supports a crucial role for the HIF-1α/PI3K/Akt/mTOR signaling pathway in mediating the Slug-regulation of E-cadherin in PCa. Nonetheless, prolonged inhibition of mTOR by rapamycin displayed diminished down-regulation of Slug, which is indicative of a resistant pathway being activated. 

As emerging evidence suggests that both the MAPK/ERK and the PI3K/Akt pathways are involved in the regulation of E-cadherin, we examined the Erk activation and are surprised to find out that bypass with Erk accounts for the resistance to mTOR inhibition for Slug. In prostate cancer cells, Slug-dependent up-regulation has been shown to down-regulate E-cadherin expression via the MAPK/ERK signaling pathway [21]. Characterized Mammalian Activated Protein Kinase (MAPK) pathway members are divided into three main subfamilies; Extracellular signal-Regulated Kinase 1/2 (ERK 1/2), p38 MAPK, and c-Jun N-terminal Kinase (JNK); Conserved through evolution, the ERK 1/2 signaling pathway is organized into a three-kinase phosphorylation cascade involving Raf (or MAPK kinase kinase), MEK (or MAPK kinase), and ERK 1/2 (or MAPK) [22,23]. Activation of ERK 1/2 in responses to an array of external stimuli can promote the expression of specific genes via phosphorylation of many transcription factors such as Elk-1 [24]. Due to the proximity to the mTOR pathway, Erk is very likely to be shunted when mTOR activity is inhibited, as shown in our study. Therefore, the inhibition of the MAPK/ERK pathways, by Cl-1040 could both used as a standalone therapy and as a sensitizer to rapamycin. 

Heat shock proteins 90 (Hsp90) consist of a highly conserved family of proteins that are required for stress tolerance in living cells. Hsp90 is a ubiquitous molecular chaperone, found to be overexpressed in a variety of cancers including prostate cancer, which is unique among molecular chaperones as the majority of its known substrates are signal transduction proteins [25]. Among its client proteins are transcription factors, cell cycle regulators, signaling kinases, mediators of apoptosis as well as steroid hormone receptors, such as the androgen receptor (AR), which have critical role in prostate carcinogenesis and in the progression to HRPC [26]. Furthermore, the AR drives growth of HRPC through a number of mechanisms that intimately rely on Hsp90 for cell survival, particularly AR overexpression and gain-of-function AR gene mutations [27]. Therefore, targeting Hsp90 is a particularly attractive anticancer approach for prostate cancer. The broad inhibitory action of Hsp90 inhibitors appears to be effective in cells expressing spliced variants of the AR that are devoid of the ligand binding domain and therefore resistant to conventional AR antagonists [28]. The first class of Hsp90 inhibitors examined was geldanamycin analogs, specifically 17-allylamino-17-demethoxy-geldanamycin (17-AAG). In our research, we found that inhibition of Hsp90 with 17-AAG diminishes Slug level, possibly caused by proteasomal degradation of AR, which led to the suppression of cell migration and invasion in both androgen-sensitive and androgen-insensitive prostate cancer cell lines.

In addition, we also observed apoptosis of prostate cancer cell by blocking the MAPK/ERK, PI3K/Akt/mTOR, and Hsp90/AR pathways. The data indicates that inhibition of ErK/mTOR/Hsp90 can cause apoptosis in cancer cells induced by down-regulation of Slug through changes in the expression of Bim. Taken together, our results indicate that the combination of Rapamycin/Cl-1040/17-AAG decreases tumor metastasis, most likely via the transcriptional suppression of Slug, which is concomitantly expressed by the activation of the MAPK/ERK, PI3K/Akt/mTOR, and Hsp90/AR. These findings indicate that Slug stays in the hub of critical pro-tumorigenic pathways and the design of combined treatments targeting Slug-related signaling cascades may have relevant implications in the prevention and treatment of this malignancy.

## References

[1] Jemal A, Tiwari RC, Murray T, Ghafoor A, Samuels A et al. (2004) Cancer statistics, 2004. CA Cancer J Clin 54: 8–29. doi:10.3322/canjclin.54.1.8. PubMed: 14974761. 14974761

[2] Jemal A, Siegel R, Ward E, Murray T, Xu J et al. (2007) Cancer statistics, 2007. CA Cancer J Clin 57: 43–66. doi:10.3322/canjclin.57.1.43. PubMed: 17237035. 17237035

[3] Chen Y, Clegg NJ, Scher HI (2009) Anti-androgens and androgen-depleting therapies in prostate cancer: new agents for an established target. Lancet Oncol 10: 981-991. doi:10.1016/S1470-2045(09)70229-3. PubMed: 19796750. 19796750 PMC2935850

[4] Holzbeierlein J, Lal P, LaTulippe E, Smith A, Satagopan J et al. (2004) Gene expression analysis of human prostate carcinoma during hormonal therapy identifies androgen-responsive genes and mechanisms of therapy resistance. Am J Pathol 164: 217-227. doi:10.1016/S0002-9440(10)63112-4. PubMed: 14695335. 14695335 PMC1602218

[5] Cooper CR, Chay CH, Gendernalik JD, Lee HL, Bhatia J et al. (2003) Stromal factors involved in prostate carcinoma metastasis to bone. Cancer 97: 739-747. doi:10.1002/cncr.11181. PubMed: 12548571. 12548571

[6] Hugo H, Ackland ML, Blick T, Lawrence MG, Clements JA et al. (2007) Epithelial--mesenchymal and mesenchymal--epithelial transitions in carcinoma progression. J Cell Physiol 213: 374-383. doi:10.1002/jcp.21223. PubMed: 17680632. 17680632

[7] Moody SE, Perez D, Pan TC, Sarkisian CJ, Portocarrero CP et al. (2005) The transcriptional repressor Snail promotes mammary tumor recurrence. Cancer Cell 8: 197-209. doi:10.1016/j.ccr.2005.07.009. PubMed: 16169465. 16169465

[8] Liu J, Uygur B, Zhang Z, Shao L, Romero D et al. (2010) Slug inhibits proliferation of human prostate cancer cells via downregulation of cyclin D1 expression. Prostate 70: 1768-1777. PubMed: 20564361. 20564361 10.1002/pros.21213 PMC2943978

[9] Nollet F, Berx G, van Roy F (1999) The role of the E-cadherin/catenin adhesion complex in the development and progression of cancer. Mol Cell Biol Res Commun 2: 77-85. doi:10.1006/mcbr.1999.0155. PubMed: 10542129. 10542129

[10] Hajra KM, Fearon ER (2002) Cadherin and catenin alterations in human cancer. Genes Chromosomes Cancer 34: 255-268. doi:10.1002/gcc.10083. PubMed: 12007186. 12007186

[11] Peinado H, Portillo F, Cano A (2004) Transcriptional regulation of cadherins during development and carcinogenesis. Int J Dev Biol 48: 365-375. doi:10.1387/ijdb.041794hp. PubMed: 15349812. 15349812

[12] Malumbres M, Pellicer A (1998) RAS pathways to cell cycle control and cell transformation. Front Biosci 3: 887-912. PubMed: 9696882. 10.2741/a331 9696882

[13] Nawshad A, Lagamba D, Polad A, Hay ED (2005) Transforming growth factor-beta signaling during epithelial-mesenchymal transformation: implications for embryogenesis and tumor metastasis. Cells Tissues Organs 179: 11-23. doi:10.1159/000084505. PubMed: 15942189. 15942189

[14] Batlle E, Sancho E, Francí C, Domínguez D, Monfar M et al. (2000) The transcription factor snail is a repressor of E-cadherin gene expression in epithelial tumour cells. Nat Cell Biol 2: 84-89. doi:10.1038/35000034. PubMed: 10655587. 10655587

[15] Cano A, Pérez-Moreno MA, Rodrigo I, Locascio A, Blanco MJ et al. (2000) The transcription factor snail controls epithelial-mesenchymal transitions by repressing E-cadherin expression. Nat Cell Biol 2: 76-83. doi:10.1038/35010506. PubMed: 10655586. 10655586

[16] McCluggage WG (2011) Morphological subtypes of ovarian carcinoma: a review with emphasis on new developments and pathogenesis. Pathology 43: 420-432. doi:10.1097/PAT.0b013e328348a6e7. PubMed: 21716157. 21716157

[17] Martin KA, Blenis J (2002) Coordinate regulation of translation by the PI 3-kinase and mTOR pathways. Adv Cancer Res 86: 1-39. doi:10.1016/S0065-230X(02)86001-8. PubMed: 12374276. 12374276

[18] Ivanova L, Butt MJ, Matsell DG (2008) Mesenchymal transition in kidney collecting duct epithelial cells. Am J Physiol Renal Physiol 294: F1238-F1248. doi:10.1152/ajprenal.00326.2007. PubMed: 18322023. 18322023

[19] Saegusa M, Hashimura M, Kuwata T, Okayasu I (2009) Requirement of the Akt/beta-catenin pathway for uterine carcinosarcoma genesis, modulating E-cadherin expression through the transactivation of slug. Am J Pathol 174: 2107-2115. doi:10.2353/ajpath.2009.081018. PubMed: 19389926. 19389926 PMC2684176

[20] Wu J, Ru NY, Zhang Y, Li Y, Wei D et al. (2011) HAb18G/CD147 promotes epithelial-mesenchymal transition through TGF-β signaling and is transcriptionally regulated by Slug. Oncogene 30: 4410-4427. doi:10.1038/onc.2011.149. PubMed: 21532623. 21532623

[21] Graham TR, Zhau HE, Odero-Marah VA, Osunkoya AO, Kimbro KS et al. (2008) Insulin-like growth factor-I-dependent up-regulation of ZEB1 drives epithelial-to-mesenchymal transition in human prostate cancer cells. Cancer Res 68: 2479-2488. doi:10.1158/0008-5472.CAN-07-2559. PubMed: 18381457. 18381457

[22] Pearson G, Robinson F, Beers Gibson T, Xu BE, Karandikar M et al. (2001) Mitogen-activated protein (MAP) kinase pathways: regulation and physiological functions. Endocr Rev 22: 153-183. doi:10.1210/er.22.2.153. PubMed: 11294822. 11294822

[23] Kolch W (2000) Meaningful relationships: the regulation of the Ras/Raf/MEK/ERK pathway by protein interactions. J Biochem 351: 289-305. doi:10.1042/0264-6021:3510289. PubMed: 11023813. PMC1221363 11023813

[24] Janknecht R, Ernst WH, Pingoud V, Nordheim A (1993) Activation of ternary complex factor Elk-1 by MAP kinases. EMBO J 12: 5097-5104. PubMed: 8262053. 8262053 10.1002/j.1460-2075.1993.tb06204.x PMC413771

[25] Csermely P, Schnaider T, Soti C, Prohászka Z, Nardai G (1998) The 90-kDa molecular chaperone family: structure, function, and clinical applications. A comprehensive review. Pharmacol Ther 79: 129-168. doi:10.1016/S0163-7258(98)00013-8. PubMed: 9749880. 9749880

[26] Chen CD, Welsbie DS, Tran C, Baek SH, Chen R et al. (2004) Molecular determinants of resistance to antiandrogen therapy. Nat Med 10: 33-39. doi:10.1038/nm972. PubMed: 14702632. 14702632

[27] Waltering KK, Urbanucci A, Visakorpi T (2012) Androgen receptor (AR) aberrations in castration-resistant prostate cancer. Mol Cell Endocrinol 360: 38-43. doi:10.1016/j.mce.2011.12.019. PubMed: 22245783. 22245783

[28] He S, Zhang C, Shafi AA, Sequeira M, Acquaviva J et al. (2013) Potent activity of the Hsp90 inhibitor ganetespib in prostate cancer cells irrespective of androgen receptor status or variant receptor expression. Int J Oncol 42: 35-43. PubMed: 23152004. 23152004 10.3892/ijo.2012.1698 PMC3583620

